# Long‐term wheel‐running prevents reduction of grip strength in type 2 diabetic rats

**DOI:** 10.14814/phy2.15046

**Published:** 2021-09-23

**Authors:** Yoshihiro Takada, Tomoko Hanaoka, Hidetaka Imagita, Toshihide Yasui, Daisuke Takeshita, Masami Abe, Shinnosuke Kawata, Taku Yamakami, Keisuke Okada, Hiroe Washio, Syunji Okuda, Akira Minematsu, Tomohiro Nakamura, Shin Terada, Takashi Yamada, Akira Nakatani, Susumu Sakata

**Affiliations:** ^1^ Division of Health Science Graduate School of Health Science Kio University Nara Japan; ^2^ Department of Human Development Graduate School of Human Development and Environment Kobe University Kobe Japan; ^3^ Department of Health and Sports Mukogawa Women's University Nishinomiya Japan; ^4^ Graduate School of Health Sciences Sapporo Medical University Sapporo Japan; ^5^ Department of Nursing School of Health Sciences Kansai University of International Studies Miki Japan; ^6^ Division of Human Sciences Faculty of Engineering Osaka Institute of Technology Osaka Japan; ^7^ Department of Life Sciences Graduate School of Arts and Sciences University of Tokyo Tokyo Japan; ^8^ Laboratory of Exercise Physiology Department of Health and Sports Science Education Nara University of Education Nara Japan; ^9^ Department of Physiology 1 Nara Medical University School of Medicine Kashihara Japan

**Keywords:** grip strength, OLETF rat, type 2 diabetes mellitus, wheel‐running exercise

## Abstract

Diabetic skeletal muscles show reduced contractile force and increased fatigability. Hands are a target for several diabetes‐induced complications. Therefore, reduced handgrip strength often occurs as a consequence of diabetes. The aim of this study was to examine whether long‐term exercise can prevent reduction of grip strength in type 2 diabetes mellitus (T2DM) model OLETF rats, and to explore the mechanisms underlying diabetes‐induced grip strength reduction. Ten 5‐week‐old OLETF rats were used as experimental animals, and five non‐diabetic LETO rats as controls of OLETF rats. Half OLETF rats performed daily voluntary wheel‐running for 17 months (OLETF + EXE), and the rest of OLETF and LETO rats were sedentary. Grip strength was higher in OLETF + EXE and LETO groups than in OLETF group. OLETF group with hyperglycemia showed an increase in HbA1c, serum TNF‐α, and muscle SERCA activity, but a decrease in circulating insulin. Each fiber area, total fiber area, and % total fiber area in type IIb fibers of extensor digitorum longus muscles were larger in OLETF + EXE and LETO groups than in OLETF group. There was a positive correlation between grip strength and the above three parameters concerning type IIb fiber area. Therefore, type IIb fiber atrophy may be the major direct cause of grip strength reduction in OLETF group, although there seems multiple etiological mechanisms. Long‐term wheel‐running may have blocked the diabetes‐induced reduction of grip strength by preventing type IIb fiber atrophy. Regular exercise may be a potent modality for preventing not only the progression of diabetes but muscle dysfunction in T2DM patients.

## Graphical abstract

Two types of hanging test were performed to evaluate not only grip ability but also muscle fatigue resistance in diabetic OLETF rats.

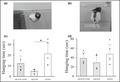

## 1 INTRODUCTION

The global prevalence of diabetes in adults has been increasing over recent decades (Cho et al., 2018; World Health Organization, 2009). The International Diabetes Federation estimated that there were 451 million people with diabetes worldwide in 2017, and the number of diabetic people was predicted to increase to 693 million by 2045 (Cho et al., 2018). Diabetes showing manifestation of chronic hyperglycemia is well‐known to increase the risk of disabling metabolic disorders including renal failure, cardiovascular disease, peripheral vascular disease, and retinopathy (World Health Organization, 2009). Moreover, diabetic skeletal muscles showed the reduced contractile force in humans (Andersen et al., 1996, 1997, 1998; Andreassen et al., 2009; Leenders et al., 2013) and rodents (Cotter et al., 1989; Fahim et al., 1998; Safwat et al., 2013; Sanchez et al., 2005; Stephenson et al., 1994), and also the increased fatigability in humans (Fritschi & Quinn, 2010) and mice (Chiu et al., 2016). Hands are a target for several diabetes‐induced complications. Diabetic patients showed a significantly higher incidence of hand abnormalities, such as Dupuytren's contracture, carpal tunnel syndrome, limited joint mobility syndrome, and flexor tenosynovitis (trigger finger), compared with non‐diabetic adults (Chammas et al., 1995; Redmond et al., 2009). Therefore, reduced handgrip strength was associated with hand disability in diabetes (Redmond et al., 2009), and was reported in patients with type 2 diabetes mellitus (T2DM) (Gundmi et al., 2018). More than 90% of the diabetic patients are T2DM, of which the onset could be prevented by physical activity (Bassuk & Manson, 2005; Grøntved et al., 2014) or healthy diet plus physical activity (The Diabetes Prevention Program Research Group, 2002).

Otsuka Long‐Evans Tokushima Fatty (OLETF) rat with the homozygously disrupted cholecystokinin type‐A receptor gene (Takiguchi et al., 1997) is a polygenic model of T2DM, which is characterized by late onset of hyperglycemia, a chronic course of disease, mild obesity, inheritance by males, hyperplastic foci of pancreatic islets, and renal complication (Kawano et al., 1992). In OLETF rats, daily voluntary wheel‐running exercise was found to have preventive effects against the development of T2DM (Mikus et al., 2010; Shima et al., 1993). In nondiabetic young OLETF rats without pancreatectomy, moreover, such a wheel‐running exercise resulted in beneficial effects on the pancreas as reflected by an increased growth of the pancreas, accompanied by increases in B‐cell mass and insulin content (Shima et al., 1997). In addition, we reported that long‐term wheel‐running exercise can prevent not only the development of T2DM but deterioration of bone properties in OLETF rats (Minematsu et al., 2017). The aims of this study were to examine whether diabetic OLETF rats show the reduced grip strength like T2DM patients, and also to examine the effects of daily physical activity on grip strength in OLETF rats. Moreover, the mechanisms underlying diabetes‐induced reduction of grip strength were explored in terms of blood biochemical and skeletal muscle histochemical properties. In this study, therefore, the voluntary wheel‐running exercise started at 1 month of age and ended at 18 months of age.

## 2 METHODS

### 2.1 Protocol approval

This study was approved by the Committee of Research Facilities of Laboratory Animal Science, Kio University and was performed in accordance with the Guide for the Care and Use of Laboratory Animals published by the US National Institutes of Health (NIH Publication No.85–23, revised in 1996).

### 2.2 Animals and experimental design

Ten 5‐week‐old male OLETF rats were used as experimental animals and five five‐week‐old male Long‐Evans Tokushima Otsuka (LETO) rats as controls of OLETF rats (Japan SLC Inc.). Both OLETF and LETO rats were established from the same colony of Long‐Evans rats (Kawano et al., 1992). The OLETF rats have been often used as an animal model of spontaneous T2DM, while the LETO rats with no manifestation of T2DM have been generally used as control animals of the experimental OLETF rats (Kawano et al., 1992). They were divided into three groups as follows; LETO, OLETF, and OLETF + EXE groups (*n* = 5 each). The OLETF + EXE rats were housed individually in a cage equipped with an exercise wheel (116 cm‐circumference, 10 cm‐inside width; Shinano), that allowed free access to the wheel, for 17 months. The number of revolutions was recorded weekly. The LETO and OLETF rats, which were housed individually in a standard cage, were regarded as a sedentary animal. Both cages were set in an animal facility where the room temperature and lighting were controlled (temperature, 23 ± 2℃; lighting, a fixed 12:12‐h light‐dark cycle). All the rats were fed a standard rodent chow (CE‐2; Clea Japan Inc.) and water ad libitum throughout the experiment. Grip strength test and hanging tests were performed within 1 month before death. At the end of 17 month‐experimental periods, the collections of blood, tibias, and skeletal muscles were performed.

### 2.3 Grip strength test

In each rat, grip strength of both fore limb and hind limb was assessed using a grip strength meter (model MK‐380 M, Muromachi Kikai Co.) as described elsewhere (McMahon et al., 2014). This meter has a wire mesh grid attached to a force transducer. Each test animal was placed carefully on the metallic mesh grid and allowed to grasp the mesh grid through all paws. The animal was then pulled back gently by holding the tail with increasing force until its grip was lost. It took 4–5 seconds to complete each measurement. This test provided the maximum grip strength force attained. Each measurement was repeated twice by the same person, and the higher force value was recorded. In the second measurement, one rat of the OLETF group disliked grasping the mesh grid, resulting in a failure in the grip strength test.

### 2.4 Mesh‐hanging tests

Two types of hanging test, that is, a reverse hanging test (Figure 4a) and a vertical hanging test (Figure 4b), were performed to evaluate not only grip ability but muscle fatigue resistance. In both hanging tests, a wire mesh grid (dimensions, 80 × 50 cm; wire mesh spacing, 12 mm; wire thickness, 1.5 mm) was used. Each test animal was allowed to grasp the mesh grid through all paws in a face‐up position in the reverse hanging test and also in a standing position in the vertical hanging test. Then we measured a grip‐holding time until the grip was released. In both hanging tests, each measurement was repeated twice by the same person, and the longer hanging time was recorded. In the second measurement, one rat of the OLETF group disliked grasping the mesh grid, resulting in a failure in both mesh‐hanging tests.

### 2.5 Collections of blood, tibias, and skeletal muscles

After 5–6 h of fasting, the rats were anesthetized with inhaled 2% (volume/volume) isoflurane on a mechanical ventilator at 12:00 p.m.–03:00 p.m. The chest was opened through a median sternotomy, and more than 10 ml of blood was obtained from the left ventricle using a 20‐guage needle. At the end of the experiment, all animals were euthanized by exsanguination under deep anesthesia with inhalation of 3% isoflurane. Serum samples, obtained from blood centrifugation at 3500 rpm for 10 min, were stored at −80℃ until biochemical analyses and enzyme‐linked immunosorbent assays (ELISA). Bilateral skeletal muscles (extensor digitorum longus (EDL), soleus (SOL), rectus femoris (RF), and biceps brachii (BB)) were obtained from the carcasses after blood collection and were weighed using a digital scale. The EDL muscles were frozen in liquid nitrogen‐cooled isopentane and stored at −80℃ until histochemical analyses. The RF muscles were frozen in liquid nitrogen and stored at −80℃ until measurements of sarcoplasmic reticulum Ca^2+^‐ATPase (SERCA) activity. The length of bilateral tibias was measured with digital caliper in order to obtain the ratio of muscle weight to tibial length (TL). When body mass differs between animals as shown by somewhat large standard deviation (SD) of body mass in OLETF group (Table 1), the ratio of muscle mass to TL is an applicable parameter to normalize muscle weight (Yin et al., 1982). Therefore, in addition to muscle mass, we herein present muscle mass normalized to TL as reported in AGE‐enriched diet‐fed mice (Egawa et al., 2017) and exercised DM rats (Farrell et al., 1999).

**TABLE 1:** Body mass, tibial length, blood glucose, and serum biochemical data

	OLETF + EXE	OLETF	LETO	Effect size
Body mass (g)	529 ± 43	496 ± 88	576 ± 18	0.286
Tibial length (mm)	45.7 ± 0.3	45.0 ± 0.8	45.8 ± 0.4	0.320
Blood glucose (mg/dl)	69 ± 26	234 ± 94*	71 ± 12	0.651
Total protein (g/dl)	5.2 ± 0.3**	5.3 ± 0.5	5.7 ± 0.2	0.229
Total cholesterol (mg/dl)	150 ± 15***	255 ± 11**	24 ± 6	0.962

Values are expressed as mean ± SD. *N* = 5 in each group.

Statistical comparisons between the groups were assessed by one‐way ANOVA followed by Fisher's LSD post hoc test.

* Significantly different from the OLETF + EXE and LETO groups (*p <* 0.01)

** Significantly different from the LETO group (*p <* 0.01)

*** Significantly different from the OLETF and LETO groups (*p <* 0.01).

### 2.6 Biochemical analyses and ELISA

Blood samples were analyzed for blood glucose concentrations and hemoglobin A1c (HbA1c) levels. Serum samples were analyzed for total protein and total cholesterol, and also with commercially available ultrasensitive ELISA kit for rat insulin (Mercodia) and ELISA strip for rat tumor necrosis factor‐α (TNF‐α; Signosis Inc.), using a microplate photometer (Multiskan FC; Thermo Fisher Scientific).

### 2.7 Histochemical analyses of EDL muscles

Histochemical analyses were performed on the frozen EDL muscle samples. Serial transverse sections (10 μm) were cut from each EDL muscle using a cryostat (CM1850, Leica microsystems) maintained at −20℃. Two serial sections were brought to room temperature and air‐dried for 30 min. One section underwent a myosin ATPase‐staining and the other a succinic dehydrogenase (SDH)‐staining, as previously reported by us (Imagita et al., 2014). Fiber type classification was performed on the basis of the staining intensities. Fiber types based on the ATPase activity were distinguished as slow‐twitch‐oxidative (Type I), fast‐twitch‐oxidative‐glycolytic (Type IIa) and fast‐twitch‐glycolytic (Type IIb), according to the classification of Peter et al. (Peter et al., 1972). To further confirm the classification of fiber types, another section was stained for the SDH activity (Nachlas et al., 1957). The cross‐sectional area (CSA) of each fiber was measured on the digitized image (1 mm^2^) of ATPase‐stained sections using a computer‐assisted image processing system (Image J version 1.46, NIH).

### 2.8 Preparations of RF muscle homogenates

The RF muscle pieces were homogenized in nine volumes of ice‐cold buffer solution (pH 7.4) consisting of 300 mM sucrose, 20 mM MOPS‐KOH, 0.0014 mM pepstatin, 0.83 mM benzamidine, 0.0022 mM leupeptin, and 0.2 mM phenylmethanesulphonyl fluoride, using a hand‐held glass homogenizer. The homogenates were then centrifuged at 2000 *g* for 20 min (2℃). The supernatant thus obtained was quickly frozen in liquid nitrogen and stored at −80℃ for the measurements of SERCA activity. The protein concentrations were determined by the method of Bradford (Bradford, 1976) using bovine serum albumin as a standard.

### 2.9 Measurements of SERCA activity in RF muscles

The SERCA activity was spectrophotometrically measured in triplicate at 37℃ as previously reported (Yamada et al., 2004). Briefly, the assay mixture (pH 7.5) contained 20 mM HEPES, 1 mM EGTA, 200 mM KCl, 15 mM MgCl_2_, 0.8 mM CaCl_2_, 10 mM sodium azide, 0.4 mM NADH, 10 mM phosphoenolpyruvate, 18 U/ml pyruvate kinase, 18 U/ml lactate dehydrogenase, and 0.005% Triton X‐100. The assay mixture was incubated for 3 min after the addition of a 20 μl aliquot of the homogenate. The reaction was started by adding Mg‐ATP to give a final concentration of 4 mM. Finally, the CaCl_2_ concentration was increased to 20 mM in order to selectively inhibit SERCA activity. The remaining activity was defined as background ATPase activity. The activity of SERCA was obtained by subtracting the background ATPase activity from total ATPase activity.

### 2.10 Statistical analyses

Values were expressed as mean ± SD. Statistical comparisons between the groups were assessed by one‐way ANOVA followed up with Fisher's LSD post hoc analysis. Moreover, effect sizes are presented as η^2^ in ANOVA of all groups. In changes in weekly running distance with aging, significant differences between the first week value and the latter values were determined using repeated‐measures ANOVA followed up with Sidak post hoc analysis. In addition, Pearson's correlation coefficients were determined to examine the relationship between grip strength and other measured parameters. All statistical analyses were performed using the Excel Statistics software (Excel 2012 version 1.08 for Windows; Social Survey Research Information Co., Ltd.,). A *P* value less than 0.05 was considered statistically significant.

## 3 RESULTS

### 3.1 Final body mass, final TL, weekly running distance, diabetes indices, and serum biochemical data

**FIGURE 1:**
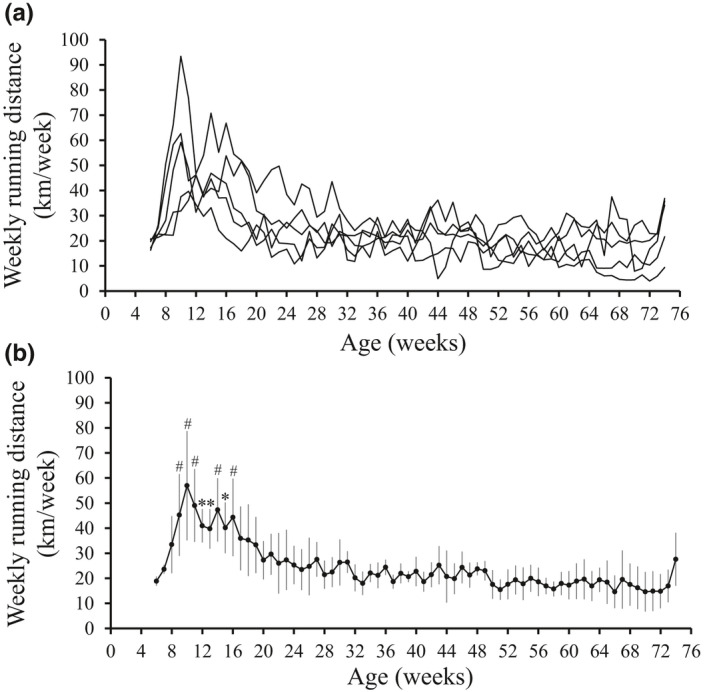
Weekly running distance. Five OLETF rats performed daily voluntary wheel‐running for a 17‐month period from age 5 weeks through age 74 weeks. (a) Individual changes in weekly running distance with aging (*n* = 5). (b) Changes in the mean weekly running distance with aging. Values are means ± SD (*n* = 5). Significant differences between the first week value and the latter values were determined using repeated‐measures ANOVA followed up with Sidak post hoc analysis. The overall *p* value obtained by repeated‐measures ANOVA is below 0.001. *Significantly different from the first week value (*p *< 0.01). ^#^Significantly different from the first week value (*p *< 0.001)

**FIGURE 2:**
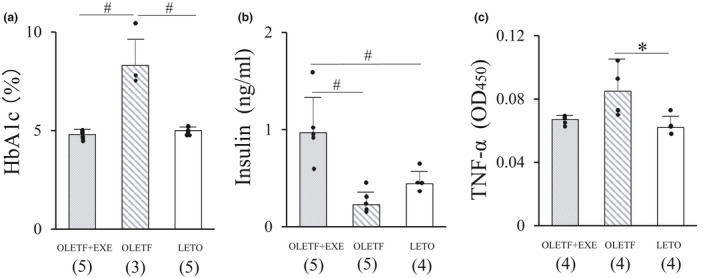
Serum levels of hemoglobin A1c (a), insulin (b) and tumor necrosis factor‐α (c) in three groups. Values are means + SD. Statistical comparisons between the groups were assessed by one‐way ANOVA followed up with Fisher's LSD post hoc analysis. *Significant difference (*p *< 0.05). ^#^Significant difference (*p *< 0.01). Symbols represent data from individual animals, and the number in the parentheses represents the number of examined animals. Effect sizes; HbA1c, 0.840; insulin, 0.699; TNF‐α, 0.374. HbA1c, hemoglobin A1c; TNF‐α, tumor necrosis factor‐α

At the end of the experiment, there was no statistically significant difference in final body mass and TL between the groups (Table 1). In the OLETF + EXE rats, there was a considerable individual difference in changes of weekly running distance with aging (Figure 1a). As shown in Figure 1b, the mean weekly running distance sharply increased from age 5–6 weeks (18.9 ± 1.8 km/week) through age 9–10 weeks (56.9 ± 21.8 km/week), and thereafter gradually declined to 18.0 ± 4.7 km/week at age 32–33 weeks, after which the running distance was similar to that of the first week for the running (age 5–6 weeks). The mean total running distance during the experimental period of 17 months was 1667 ± 375 km, with a wide range from 1171 to 2219 km. Diabetes indices, that is, blood glucose and HbA1c levels, were much lower in the OLETF + EXE and LETO groups than in the OLETF group (Table 1 and Figure 2a). Thus the OLETF + EXE and LETO rats showed similar diabetes indices. Serum concentration of total protein was lower in the OLETF + EXE group than in the LETO group (Table 1). Although the OLETF group showed the highest levels of total cholesterol in the three groups, the OLETF + EXE group showed the intermediate total cholesterol levels between the LETO group's and OLETF group's levels (Table 1). Circulating insulin levels were significantly higher in the OLETF + EXE group than in the OLETF and LETO groups (Figure 2b). The OLETF group indicated the highest levels of TNF‐α among the three groups (Figure 2c).

### 3.2 Grip strength and hanging capacity

**FIGURE 3:**
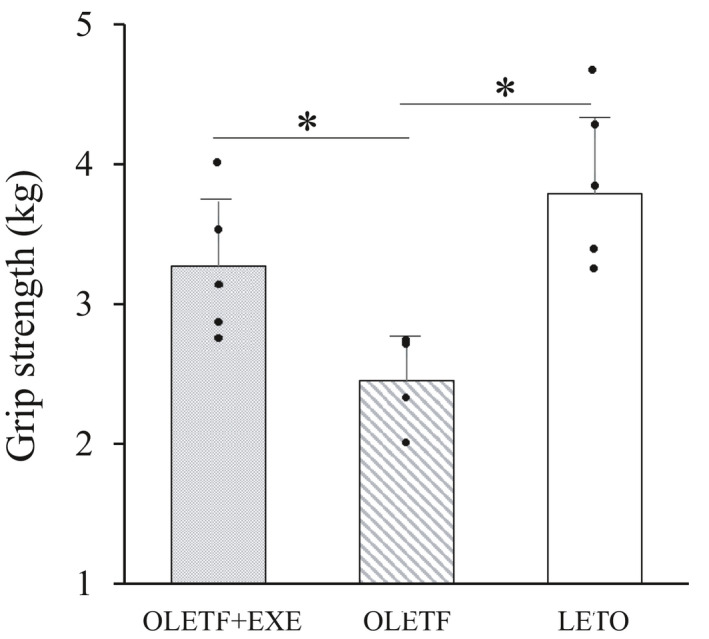
Grip strength in three groups. Values are means + SD. Statistical comparisons between the groups were assessed by one‐way ANOVA followed up with Fisher's LSD post hoc analysis. *Significant difference (*p *< 0.05). Symbols represent data from individual animals. *N* = 4 in the OLETF group, and *n* = 5 in the OLETF+EXE and LETO groups. Effect size is 0.702

**FIGURE 4:**
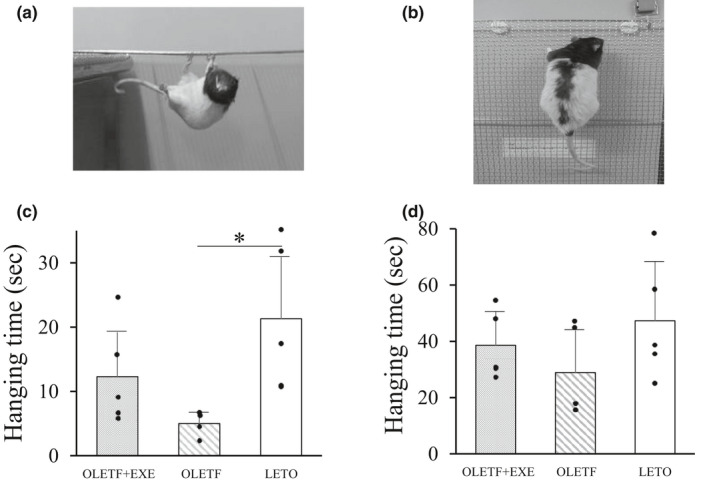
Photograph of animal in the reverse (a) and vertical (b) hanging tests, and hanging time in the reverse (c) and vertical (d) hanging tests of three groups. Values are means + SD. Statistical comparisons between the groups were assessed by one‐way ANOVA followed up with Fisher's LSD post hoc analysis. *Significant difference (*p *< 0.05). Symbols represent data from individual animals. In both hanging tests, *n* = 4 in the OLETF group, and *n* = 5 in the OLETF + EXE and LETO groups. Effect sizes; reverse hanging test, 0.426; vertical hanging test, 0.200

The grip strength was significantly higher in the OLETF + EXE and LETO groups than in the OLETF group (Figure 3). A similar pattern was observed in both the reverse and vertical hanging tests (Figure 4c,d). The OLETF group showed the shortest hanging time among the three groups.

### 3.3 Muscle mass and muscle mass/TL ratio

Table 2 indicates the muscle mass and muscle mass/TL ratio in the EDL, SOL, BB and RF muscles. Although the OLETF + EXE and OLETF rats had similar muscle mass and muscle mass/TL ratio in four kinds of muscles, the LETO rats had the increased muscle mass and muscle mass/TL ratio in the EDL, SOL, and BB muscles, compared with the OLETF + EXE and OLETF rats.

**TABLE 2:** Muscle mass and the ratio of muscle weight to tibial length

	OLETF + EXE	OLETF	LETO	Effect size
EDL mass (mg)	220.5 ± 6.7**	216.9 ± 28.8**	257.9 ± 8.8	0.577
EDL mass/TL (mg/mm)	4.83 ± 0.15**	4.82 ± 0.50**	5.63 ± 0.19	0.585
SOL mass (mg)	188.9 ± 20.4*	180.3 ± 34.6**	233.6 ± 15.0	0.527
SOL mass/TL (mg/mm)	4.14 ± 0.42*	4.00 ± 0.62**	5.10 ± 0.31	0.522
BB mass (mg)	283.5 ± 8.7*	268.9 ± 33.7**	322.9 ± 21.4	0.539
BB mass/TL (mg/mm)	6.21 ± 0.15*	5.97 ± 0.58**	7.04 ± 0.38	0.560
RF mass (mg)	1480.2 ± 84.7	1440.0 ± 180.5	1630.2 ± 133.8	0.303
RF mass/TL (mg/mm)	32.4 ± 1.7	32.0 ± 3.1	35.6 ± 2.8	0.273

Values are expressed as mean ± SD. *N* = 5 in each group.

Statistical comparisons between the groups were assessed by one‐way ANOVA followed by Fisher's LSD post hoc test.

Abbreviations: BB, biceps brachii; EDL, extensor digitorum longus; RF, rectus femoris; SOL, soleus; TL, tibial length.

* Significantly different from the LETO group (*p *< 0.05)

** Significantly different from the LETO group (*p *< 0.01).

### 3.4 Characterization of EDL muscle fibers

**FIGURE 5:**
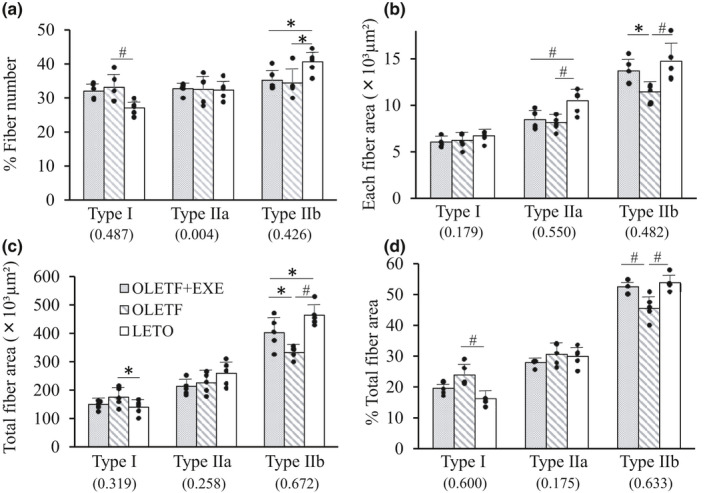
Characterization of extensor digitorum longus muscle fibers in the three groups. Percent fiber number (a), each fiber area (b), total fiber area (c), and percent total fiber area (d) in the type I, type IIa, and type IIb fibers. Grey bars, the OLETF + EXE group; striped bars, the OLETF group; white bars, the LETO group. Values are means + SD. Statistical comparisons between the groups were assessed by one‐way ANOVA followed up with Fisher's LSD post hoc analysis. *Significant difference (*p *< 0.05). ^#^Significant difference (*p *< 0.01). Symbols represent data from individual animals, and the value in the parentheses represents effect size. *N* = 5 in each group

In three groups, we identified the fiber type of EDL muscle associated with grip of hind limb (Figure 5). In the type I fibers, the OLETF group showed an increase in % fiber number (Figure 5a), total fiber CSA (Figure 5c) and % total fiber CSA (Figure 5d) compared with the LETO group. To the contrary, in the type IIb fibers, the OLETF and OLETF + EXE groups showed a decrease in % fiber number (Figure 5a) compared with the LETO group, and also the OLETF group showed a decrease in each fiber CSA (Figure 5b), total fiber CSA (Figure 5c) and % total fiber CSA (Figure 5d) compared with the OLETF + EXE and LETO groups. Moreover, in the type IIa fibers, the OLETF and OLETF + EXE groups exhibited a decrease in each fiber CSA compared with the LETO group (Figure 5b). As shown in Figure 5b,d, there was no significant difference in each fiber CSA and % total fiber CSA of the type IIb fibers between the OLETF + EXE and LETO groups.

### 3.5 SERCA activity in RF muscles

**FIGURE 6:**
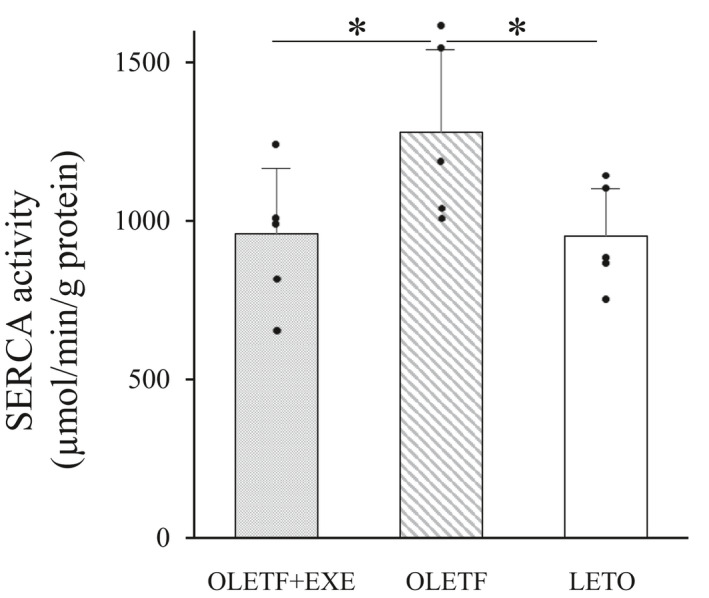
Sarcoplasmic reticulum Ca^2+^‐ATPase activity in the rectus femoris muscles from three groups. Values are means + SD. Statistical comparisons between the groups were assessed by one‐way ANOVA followed up with Fisher's LSD post hoc analysis. *Significant difference (*p *< 0.05). Symbols represent data from individual animals. *N* = 5 in each group. SERCA, sarcoplasmic reticulum Ca^2+^‐ATPase. Effect size is 0.351

In the RF muscles, the SERCA activity was significantly higher in the OLETF group than in the OLETF + EXE and LETO groups (Figure 6). The mean SERCA activity in the OLETF + EXE group was similar to that of the LETO group.

### 3.6 Correlations between grip strength and various parameters

We examined correlations between grip strength and various measured parameters, using the combined data from the three groups (Table 3). There was a good negative correlation between grip strength and blood glucose, HbA1c, total cholesterol, TNF‐α, % fiber number of type I, or % total fiber CSA of type I and also a negative, but not significant (*p* = 0.075), correlation between grip strength and SERCA activity. To the contrary, there was a good positive correlation between grip strength and muscle mass (EDL, SOL and BB), each fiber CSA of type IIa or IIb fibers, total fiber CSA of type IIa or IIb fibers, or % total fiber CSA of type IIb fibers.

**TABLE 3:** Correlations between grip strength and various parameters

	r	*p*
Body mass	0.448	0.123
Blood glucose	−0.819	0.001
Hb A1c	−0.695	0.012
Total protein	0.001	0.998
Total cholesterol	−0.775	0.002
Insulin	0.215	0.502
TNF‐α	−0.684	0.020
SERCA activity	−0.510	0.075
EDL mass	0.633	0.020
SOL mass	0.663	0.013
BB mass	0.600	0.030
RF mass	0.466	0.109
%FN‐I	−0.636	0.019
%FN‐IIa	0.483	0.095
%FN‐IIb	0.285	0.345
FA‐I	0.379	0.201
FA‐IIa	0.566	0.044
FA‐IIb	0.773	0.002
TFA‐I	−0.408	0.167
TFA‐IIa	0.560	0.047
TFA‐IIb	0.752	0.003
%TFA‐I	−0.707	0.007
%TFA‐IIa	0.169	0.582
%TFA‐IIb	0.568	0.043

Pearson's correlation coefficients were calculated to determine the relation between grip strength and other measured parameters.

Abbreviations: %TFA, percent total fiber area; BB, biceps brachii; EDL, extensor digitorum longus; FA, fiber area; FN, fiber number; Hb A1c, hemoglobin A1c; RF, rectus femoris; SERCA, sarcoplasmic reticulum Ca^2+^‐ATPase; SOL, soleus; TFA, total fiber area; TNF‐α, tumor necrosis factor‐α.

## 4 DISCUSSION

In this study, we have examined the effects of long‐term wheel‐running exercise not only on grip strength/hanging capacity but on blood biochemical/muscle histochemical properties in the OLETF rats. Table 4 summarizes the comparison between the OLETF + EXE rats and the sedentary OLETF or LETO rats in terms of various parameters. Our findings are as follows: (a) Grip strength as well as hanging capacity was higher in the OLETF + EXE and LETO rats than in the OLETF rats. (b) Blood glucose, HbA1c, total cholesterol, and TNF‐α levels were lower in the OLETF + EXE and LETO rats than in the OLETF rats. To the contrary, serum insulin levels were higher in the OLETF + EXE rats than in the OLETF and LETO rats. (c) Muscle mass and muscle mass/TL ratio were smaller in the OLETF + EXE and OLETF rats than in the LETO rats. (d) Each fiber CSA, total fiber CSA and % total fiber CSA in the type IIb fibers of EDL muscles were larger in the OLETF + EXE and LETO rats than in the OLETF rats. (e) SERCA activity in the muscles was lower in the OLETF + EXE and LETO rats than in the OLETF rats. (f) Grip strength was correlated negatively with blood glucose, HbA1c, total cholesterol, TNF‐α, % fiber number of type I fibers, % total fiber CSA of type I fibers and SERCA activity and also positively with muscle mass, each fiber CSA of type IIa or IIb fibers, total fiber CSA of type IIa or IIb fibers and % total fiber CSA of type IIb fibers. Thus, in the OLETF rats, long‐term wheel‐running could prevent not only the development of T2DM but the reduction of grip strength. To the best of our knowledge, this is the first study to report not only the effects of long‐term exercise on grip strength/hanging capacity but the correlations between grip strength and blood biochemical/muscle histochemical properties in the OLETF rats. There seems to be several possible mechanisms underlying diabetes‐induced reduction of grip strength in the OLETF rats as described below.

**TABLE 4:** OLETF + EXE group is compared with the OLETF and LETO groups in terms of various parameters

Measurements	Vs. OLETF group	Vs. LETO group
Body size parameters		
Body mass	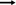	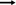
Tibial length	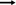	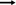
Diabetic parameters		
Blood glucose	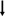	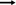
HbA1c	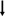	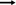
Serum biochemical parameters		
Insulin	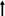	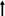
TNF‐α	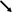	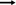
Total protein	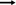	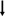
Total cholesterol	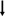	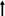
Grip strength and hanging capacity		
Grip strength	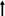	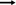
Reverse hanging capacity	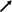	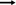
Vertical hanging capacity	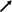	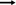
Muscle mass and SERCA activity		
Muscle mass (EDL, SOL, BB)	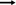	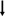
Muscle mass/TL (EDL, SOL, BB)	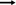	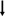
SERCA activity (RF)	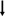	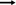
EDL muscle fiber properties		
%FN‐I	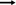	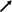
%FN‐IIa	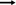	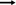
%FN‐IIb	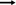	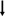
FA‐I	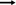	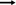
FA‐IIa	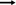	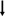
FA‐IIb	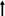	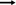
TFA‐I	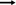	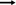
TFA‐IIa	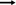	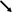
TFA‐IIb	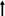	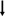
%TFA‐I	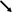	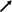
%TFA‐IIa	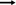	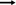
%TFA‐IIb	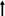	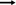

HbA1c, hemoglobin A1c; TNF‐α, tumor necrosis factor‐α; SERCA, sarcoplasmic reticulum Ca^2+^‐ATPase; EDL, extensor digitorum longus; SOL, soleus; BB, biceps brachii; RF, rectus femoris; TL, tibial length; FN, fiber number; FA, fiber area; TFA, total fiber area.

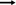
, no difference; 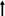, significant increase; 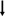, significant decrease; 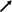, increase without statistical significance; 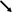, decrease without statistical significance.

First, the grip strength appears to depend primarily upon the wet muscle mass because there was a positive correlation between the grip strength and the muscle mass (Table 3). Likewise, in a magnetic resonance imaging study on DM patients, the maximal isokinetic muscle strength of ankle dorsal/plantar flexors was related to the muscle volume (Andersen et al., 1997). Moreover, in streptozotocin (STZ)‐induced DM rats with the decreased EDL muscle mass, the isometric contractile force of EDL (Cotter et al., 1989) or of single EDL fibers (Stephenson et al., 1994) was significantly lower than that of non‐DM rats.

The present OLETF group showed the decreased levels of circulating insulin, which is well known to regulate muscle protein synthesis/proteolysis. In alloxan‐induced DM rats, the protein synthesis of fast‐type muscles (gastrocnemius (GA) and tibialis anterior (TA)) was impaired by the block of peptide‐chain initiation, which was caused by the insulin deficiency (Flaim et al., 1980). STZ‐induced DM rats with reduced plasma insulin levels showed the enhanced degradation of myofibrillar protein (Smith et al., 1989). Both the impaired muscle protein synthesis and enhanced myofibrillar protein degradation in DM state could be restored by the exogenous insulin treatment (Flaim et al., 1980; Smith et al., 1989). Therefore, in our OLETF group, the decreased muscle mass may have been due to the insulin deficiency.

Circulating insulin‐like growth factor I (IGF‐I), which stimulates muscle protein synthesis like insulin, also declined in DM rats (Derakhshanian et al., 2017; Farrell et al., 1999). In fasted mice, administration of IGF‐I or insulin induced a 52%–65% increase in protein synthesis of fast‐type muscles (GA and plantaris), but only a 11%–25% increase in that of slow‐type SOL muscle (Bark et al., 1998). Furthermore, IGF‐I overexpression in the transgenic murine muscle led to myofiber hypertrophy (Coleman et al., 1995), and local IGF‐I infusion into the skeletal muscles resulted in muscle hypertrophy (Adams & McCue, 1998). Insulin and IGF‐I are thought to promote protein synthesis via an activated PI3K/Akt/mTOR signaling pathway, resulting in myofiber/muscle hypertrophy (Bassel‐Duby & Olson, 2006). On the other hand, in STZ‐induced DM rats, the decline in circulating IGF‐I induced the gene expression of atrogin‐1/MAFbx, a muscle‐specific ubiquitin‐ligase required for muscle atrophy, resulting in the loss of muscle mass (Dehoux et al., 2004). In DM rats, chronic resistance exercise induced the increase in both plasma IGF‐I levels and muscle protein synthesis, leading to a rise in the muscle mass/TL ratio (Farrell et al., 1999). In the current study, however, wheel‐running exercise failed in increasing the muscle mass, although it led to the increase in serum insulin concentrations. At present, the cause of such a discrepancy concerning the exercise effect on DM muscle mass remains to be resolved.

Second, the grip strength also appears to depend primarily upon type IIb fiber CSA in EDL muscles, because the grip strength correlated fairly well with each fiber CSA, total fiber CSA and % total fiber CSA of type IIb fibers. Similarly, a positive correlation was found between type II fiber CSA in the vastus lateralis (VL) muscle and the maximum extension strength of leg muscles in T2DM patients (Leenders et al., 2013). The present OLETF group showed the reduction in both grip strength and EDL type IIb fiber CSA. Likewise, forelimb grip strength and fiber CSA of fast glycolytic TA muscle were reduced in *db/db* mice, of which fast‐type muscle mass was decreased (Ostler et al., 2014). In STZ‐induced DM rats, moreover, EDL muscles with 44% reduction in total type IIb fiber CSA indicated a decrease in the maximum isometric twitch tension (Cotter et al., 1989). In addition, in insulin‐treated DM rats, the decrease in peak force of single type IIb fiber from EDL muscles was due to type IIb fiber atrophy (Sanchez et al., 2005). In view of the above‐mentioned observations, it seems most likely that the reduced grip strength in the present OLETF group is mainly ascribable to fast‐type muscle atrophy accompanied by type IIb fiber atrophy.

Circulating corticosterone levels were elevated in STZ‐induced DM rats (Smith et al., 1989) and *db/db* mice (Ostler et al., 2014). Glucocorticoid treatment resulted in a 25% reduction in fast‐type muscle mass by the decreased protein synthesis, but did not affect the slow‐type SOL mass in normal rats (Rannels & Jefferson, 1980). Moreover, type IIb fiber CSA in the corticosteroid‐treated rat diaphragm was reduced by 51%, whereas type I and IIa fiber CSAs were unaffected (Dekhuijzen et al., 1995). Overexpression of IGF‐I in TA muscle prevented muscle/myofiber atrophy in glucocorticoid‐treated rats (Schakman et al., 2005). Therefore, glucocorticoid‐induced muscle/myofiber atrophy appears to be caused by downregulating the IGF‐I‐signaling pathways (Schakman et al., 2009). Exercise could induce not only the increase in circulating IGF‐I levels (Farrell et al., 1999) but the decrease in diabetes‐induced serum corticosterone levels (Hwang et al., 2011) in DM rats, resulting in the prevention of muscle atrophy (Farrell et al., 1999). Although the present wheel‐running exercise caused the decrease in % type IIb fiber number and the increase, but not significant, in % type I fiber number in agreement with the observation that 45‐day wheel‐running exercise resulted in reduced proportions of type IIb fibers in EDL muscles of normal rats (Kriketos et al., 1995), it prevented the decrease in type IIb fiber CSA in EDL muscles. Lifelong (21 months) wheel‐running exercise combined with mild caloric restriction resulted in a significant increase in IGF‐I protein levels and fiber CSA in rat fast‐type plantaris muscles (Kim et al., 2008). In view of these results, our long‐term (17 months) wheel‐running exercise may have prevented type IIb fiber atrophy by the enhanced IGF‐I expression of EDL muscles, increased circulating insulin, and decreased circulating corticosterone levels.

Third, DM‐induced reduction of grip strength may be attributable to inflammation, because the serum TNF‐α concentrations were increased in the present OLETF group and were correlated negatively with grip strength. Likewise, circulating levels of pro‐inflammatory cytokines, TNF‐α and interleukin (IL)‐6, were increased in older T2DM patients with excessive loss of skeletal muscle mass (Park et al., 2009), and such higher TNF‐α/IL‐6 levels were associated with lower grip strength in elderly persons (Visser et al., 2002). The TNF‐α caused skeletal muscle atrophy via the IKK*β*/ NF‐*κ*B/MURF1 signaling pathway (Cai et al., 2004). In 26‐month‐old rats, moreover, the superficial VL muscles composed predominantly of type II fast fibers exhibited an increase in TNF‐α expression and TNF‐α‐induced apoptosis compared with the SOL muscles composed predominantly of type I slow fibers, suggesting TNF‐α signal transduction specific to type II fast fibers (Phillips & Leeuwenburgh, 2005). Thus, TNF‐α appears to be a powerful fast‐type fiber‐wasting cytokine.

In this study, the mean concentration of serum TNF‐α was lower in the OLETF+EXE group than in the OLETF group. Similarly, in patients with T2DM and the metabolic syndrome, aerobic plus resistance‐combined physical exercise for 1 year decreased serum levels of pro‐inflammatory cytokines TNF‐α, IL‐1β, IL‐6, and interferon‐γ, but reversely increased serum levels of anti‐inflammatory cytokines IL‐4 and IL‐10, thus indicating that exercise has a full anti‐inflammatory effect in T2DM (Balducci et al., 2010). In addition, 8 weeks of aerobic exercise increased the protein content of NF‐*κ*B‐inhibitor I*κ*B and reduced TNF‐α protein content in skeletal muscle of T2DM patients, suggesting that NF‐*κ*B signaling is downregulated by exercise (Sriwijitkamol et al., 2006). Thus, exercise may prevent diabetes‐induced muscle atrophy by downregulating NF‐*κ*B signaling in the potential inflammatory pathway.

Fourth, non‐enzymatic glycosylation (glycation) of grip‐associated proteins may cause the attenuation of grip strength, because the glycosylated hemoglobin, that is, HbA1c, were elevated in the present OLETF group and correlated negatively with grip strength. Such a negative correlation between HbA1c and grip strength was also found in patients with T2DM (Leenders et al., 2013). Skeletal muscle myosin from DM patients was more glycosylated and had lower Ca^2+^‐ATPase activity compared with control myosin of healthy subjects (Syrovỳ & Hodnỳ, 1992). In rat myofibrillar preparations, as myofibrillar proteins were glycated by incubation with ribose, Mg^2+^‐activated ATPase activity of myofibrils was lowered (Syrovỳ & Hodnỳ, 1993). Similarly, in single fibers from rat skinned EDL muscles exposed to glucose‐6‐phosphate, the maximum Ca^2+^‐activated force per CSA and Mg^2+^‐activated ATPase activity were significantly reduced (Patterson et al., 2000). Moreover, rat skeletal muscle myosin was incubated with glucose and subsequently analyzed for structural and functional modifications by matrix‐assisted laser desorption/ionization (MALDI) mass spectrometry and a single‐fiber in vitro motility assay (Ramamurthy et al., 2001). Glycation‐related structural alterations, revealed by MALDI spectra, were paralleled by a significant reduction in the in vitro motility speed, suggesting a structural‐related decline in myosin mechanics in response to glucose exposure (Ramamurthy et al., 2001).

In type 1 DM (T1DM) patients, skin collagen was glycated with the duration of diabetes, and there was a positive correlation between glycated collagen levels and finger‐joint stiffness (Monnier et al., 1986). Moreover, the glycated collagen matrix exhibited a decreased elasticity and increased toughness compared with the non‐glycated collagen matrix (Liao et al., 2009). In addition, fibroblast‐mediated contraction of the glycated collagen matrix was reduced as compared with that of the non‐glycated collagen matrix (Liao et al., 2009). Thus, the glycated collagen, too, may contribute to reduced grip strength in DM.

Advanced glycation end‐products (AGEs) accumulated in skin correlated negatively with grip strength in adult men (Momma et al., 2011). AGEs, accumulated in rat EDL myofibers with aging, selectively modified actin and several metabolic enzymes involved in energy production, like creatine kinase (CK) and β‐enolase (Snow et al., 2007). Therefore, such AGE‐induced posttranslational modifications of contraction‐associated proteins are considered as one of the causes of muscle dysfunction. In fact, mice fed an AGE‐enriched diet for 16 weeks exhibited skeletal muscle dysfunction, including the reduction in grip strength, fatigue resistance and in vitro muscle force production (Egawa et al., 2017). In addition, the AGEs accumulation in the muscles of STZ‐induced DM mice and a DM patient was associated with muscle atrophy and muscle dysfunction via a putative mechanism of the RAGE‐mediated, AMPK‐downregulated, Akt signaling pathway (Chiu et al., 2016).

Fifth, grip strength weakness in DM may be associated with the downregulation of SERCA gene expression, because vitamin D receptor knockout mice and diet‐induced vitamin D deficient mice showed the reduction in both grip strength and SERCA gene expression (Girgis et al., 2015). Skeletal muscle contraction is tightly associated with excitation‐contraction coupling in myofibers. The sarcoplasmic reticulum (SR), which releases Ca^2+^ via the ryanodine receptor during contraction and, during relaxation, takes it up by the SERCA pump, plays a major role in intracellular Ca^2+^ handling. Thus, muscle contraction/relaxation function is critically dependent on effective Ca^2+^ handling. For instance, in DM rats 8 weeks after the STZ‐injection, SERCA activity and isometric contractile force of the skeletal muscles are increased (Ganguly et al., 1986). To the contrary, high‐fat diet‐fed T2DM rats 12 weeks after the STZ‐injection showed the decrease in both SERCA gene expression and contractile performance of GA muscles (Safwat et al., 2013). In these rats, however, adiponectin gene therapy and/or swimming exercise induced the marked SERCA gene expression, and consequently improved muscle contractility (Safwat et al., 2013). Such a muscle dysfunction in T2DM appears to be mediated via abnormal Ca^2+^ handling due to impaired SERCA expression. In our OLETF group, grip strength was much reduced as compared with two other groups, but, contrary to our expectation, SERCA activity in the skeletal muscle was enhanced as reported in STZ‐induced DM rats (Ganguly et al., 1986) and T1DM patients (Harmer et al., 2014). Unphosphorylated phospholamban (PLB) inhibits the SERCA activity by lowering its apparent Ca^2+^ affinity. Phosphorylation of PLB by Ca^2+^/calmodulin kinase II relieves this inhibition and elicits Ca^2+^ uptake activity of SERCA (Vangheluwe et al., 2005). Calmodulin levels were elevated in the GA muscle from STZ‐induced DM mice and diabetic *db/db* mice (Morley et al., 1982). Therefore, it seems likely that the enhanced SERCA activity reported herein is ascribable to PLB phosphorylation induced by increased Ca^2+^/calmodulin kinase II. Similar to the reverse relation between SERCA activity and grip strength in our OLETF rats, hindlimb unweighting caused dramatic increases in SERCA1a activity in rat skeletal muscle, of which contractile performance deteriorated (Schulte et al., 1993). Such an enhanced SERCA activity may be a compensative response to the reduced muscle contractility.

Sixth, the reduced grip strength in DM state may be caused by failure in neuromuscular signal transmission, since the muscle‐nerve preparations of T2DM *db/db* mice displayed reduced axonal excitability and force deficit with indirect stimulation via the nerve compared with the control preparations (Bayley et al., 2016). This is supported by a negative correlation between isokinetic muscle strength at the ankle or knee and the neuropathy rank‐sum score in T1DM patients (Andersen et al., 1996). Such a muscle strength may be impaired by incomplete re‐innervation following axonal loss in DM patients with neuropathy (Andersen et al., 1998). Moreover, in DM patients, the gene expression of neurotrophin‐3 was reduced in striated muscles and was related to muscle weakness and neuropathy, suggesting that incomplete re‐innervation in DM state is caused by neurotrophin‐3 deficiency (Andreassen et al., 2009). On the other hand, muscles of STZ‐induced DM mice showed the desensitization of nicotinic acetylcholine receptor channels (Nojima et al., 1995). Moreover, such DM mice exhibited neuromuscular ultrastructure changes including severe loss of synaptic vesicles and degeneration of mitochondria at neuromuscular junctions, and, at the muscle level, swollen mitochondria with disorganization of their cristae and disruption of the t‐tubules (Fahim et al., 1998).

Last, the impaired grip strength in our OLETF group may be attributed to the defective energy transduction and/or the shortage of energy reserves in diabetic muscles. The cytosolic CK plays a pivotal role in anaerobic energy transduction. The cytosolic ATP concentration is maintained relatively constant primarily via a high‐energy phosphate transfer by CK to form ATP, that is, the rapid enzymatic transfer of a phosphate from phosphocreatine (PCr) to ADP to form ATP is catalyzed by CK. Thus, PCr can serve as a high‐energy reservoir to sustain the muscle contraction, especially in anaerobic quick intensive exercise such as grip strength test. In STZ‐induced DM rats, CK activity was decreased in SOL (Su et al., 1992) and EDL muscles (Xin et al., 1999), and, consequently, the intracellular ATP levels were reduced in diabetic muscle (Moore et al., 1983). Indeed, single myofibers from CK‐deficient mice displayed the reduced tetanic force during initial unfatigued period of electrical stimulation (Dahlstedt et al., 2000). A study using MRI spectroscopy demonstrated that the intramuscular PCr/inorganic phosphate (Pi) ratio, which is considered as an index of muscle energy reserve state, is lower in neuropathic DM patients than in the healthy subjects (Dinh et al., 2009). In fact, PCr‐depleted skeletal muscles exhibited the isometric contractile dysfunction in rats fed a diet containing the creatine analogue, β‐guanidinopropionic acid (Petrofsky & Fitch, 1980).

In view of these facts, the reduced grip strength in our OLETF group may have been caused by the above‐mentioned multiple mechanisms derived from DM‐related disorder or diabetic complications, though type IIb fiber atrophy may be the major direct cause of reduced grip strength.

In an attempt to evaluate muscle fatigue resistance, two types of mesh‐hanging test were carried out in this study. The diabetic OLETF group, as well as dietary AGE‐fed mice in the wire‐hanging test (Egawa et al., 2017), showed the shortest hanging time, indicating the reduced fatigue resistance. The rise in Pi resulting from the breakdown of PCr is thought to play a central role in skeletal muscle fatigue, of which the underlying mechanisms are as follows: increasing myoplasmic Pi reduces active crossbridge force, myofibrillar Ca^2+^ sensitivity, and SR Ca^2+^ release, leading to reduced force production (Allen & Westerblad, 2001). In the OLETF rats under the present hanging tests, this PCr breakdown in myoplasm would occur quickly, and, consequently, increasing myoplasmic Pi would cause the earlier grip‐release. Microinjection of CK into CK‐deficient mouse muscle fibers markedly restored both tetanic force and free myoplasmic Ca^2+^ concentration during a period of high‐intensity electrical stimulation, indicating that CK is important for preventing fatigue during high‐intensity stimulation (Dahlstedt et al., 2003). As mentioned above, CK activity was decreased in diabetic skeletal muscles (Su et al., 1992; Xin et al., 1999). Therefore, the increased muscle fatigability in our diabetic OLETF rats may have been due to decreased CK activity in muscle fibers.

This study has some limitations. First, sample size was small because the Kio University restricted the number of experimental animals based on 3 R principles (reduction, replacement, and refinement). Generalizing our data may be limited because of this modest sample size. Therefore, not only *p* values but effect sizes are presented herein to confirm whether the difference between groups is significant or not. The statistical analyses seem valid based on the values of effect size. However, studies with a larger sample size are required to further confirm our findings. Second, this study had neither baseline data of blood biomarkers and muscle morphological characteristics in young rats nor data of in vivo muscle function examined at regular intervals throughout the experimental period. These data will bring forth an optimal exercise "dose" for improving muscle function. Third, examination of the isometric contractile force in isolated EDL muscles and single EDL myofibers will further support the present positive effects of exercise on in vivo muscle function. Fourth, we have not measured muscle/myofiber atrophy‐related factors (IGF‐I and corticosterone), quick energy production‐related substances (CK and PCr), muscle force production‐related substances (AGEs and Pi), and neuropathy‐related neurotrophin‐3. The measurements of these substances are needed to better understand the overall picture of the mechanisms underlying reduced grip strength in DM state. Fifth, similar studies using other DM animal models, for example, STZ‐induced DM rodents, T2DM *db/db* mice, etc., are required for further confirming the data presented in this study. Sixth, rearing the rats individually for the long period of 17 months may have put the rats under some stress, because the rat is a sociable animal. Although the circulating adrenocorticotropic hormone and cortisol levels have not been measured in this study, all rats showed normal behavior and no hair loss, suggesting that they may have suffered from little or no stress. Finally, further studies, in which adult diabetic OLETF rats are subjected to exercise, are needed to examine improvable effects, but not preventive effects herein, of exercise on DM‐induced attenuation of grip strength.

## 5 CONCLUSION

The present results indicate that the long‐term wheel‐running can prevent the reduction of grip strength in the T2DM model OLETF rats. This exercise could also prevent the type IIb fiber atrophy, caused by diabetes, in EDL muscles. In addition, there was a good positive correlation between grip strength and CSA, total CSA or % total CSA in type IIb fibers. Therefore, type IIb fiber atrophy may be the major direct cause of grip strength reduction in diabetic OLETF rats, though there seems several other etiological mechanisms involving AGE‐induced protein modifications, potential inflammatory signaling pathway, abnormal Ca^2+^ handling, incomplete neuromuscular signal transmission and impaired energy transduction/reserves. Long‐term wheel‐running may have blocked the diabetes‐induced reduction of grip strength by preventing type IIb fiber atrophy. Regular physical exercise may be a potent modality for preventing not only the progression of diabetes but muscle dysfunction, like grip strength reduction, in T2DM patients.

## CONFLICT OF INTEREST

No conflicts of interest, financial or otherwise, are declared by the authors.

## AUTHORS' CONTRIBUTIONS

Y.T., T.H., H.I., A.M., and S.S. conceived and designed research. Y.T., T.H., T. Yasui, D.T., M.A., S.K., T. Yamakami, K.O., H.W., S.O., A.M., T. Yamada, A.N., and S.S. performed experiments. Y.T., T. Yamada, A.N., and S.S. analyzed data. Y.T., H.I., T.N., S.T., T. Yamada, A.N., and S.S. interpreted results of experiments. Y.T. and S.S. prepared figures. Y.T. and S.S. drafted manuscript. Y.T., T.H., H.I., T. Yasui, D.T., M.A., S.K., T. Yamakami, K.O., H.W., S.O., A.M.,T.N., S.T., T. Yamada, A.N., and S.S. approved the final version of the manuscript.
